# Intubation practices and outcomes for patients with suspected or confirmed COVID-19: a national observational study by the Canadian COVID-19 Emergency Department Rapid Response Network (CCEDRRN)

**DOI:** 10.1007/s43678-023-00487-1

**Published:** 2023-04-05

**Authors:** Murdoch Leeies, Rhonda J. Rosychuk, Muzeen Ismath, Ke Xu, Patrick Archambault, Patrick T. Fok, Thomas Audet, Tomislav Jelic, Jake Hayward, Raoul Daoust, Kavish Chandra, Phil Davis, Justin W. Yan, Jeffrey P. Hau, Michelle Welsford, Steven C. Brooks, Corinne M. Hohl, The Canadian COVID-19 Emergency Department Rapid Response Network (CCEDRRN) investigators for the Network of Canadian Emergency Researchers, the Canadian Critical Care Trials Group

**Affiliations:** 1 Department of Emergency Medicine, University of Manitoba, Winnipeg, MB Canada; 2 Rady Faculty of Health Sciences, Section of Critical Care Medicine, University of Manitoba, Winnipeg, MB Canada; 3 Department of Pediatrics, University of Alberta, Edmonton, AB Canada; 4 Department of Emergency Medicine, University of British Columbia, Vancouver, BC Canada; 5 Department of Family Medicine and Emergency Medicine and Department of Anesthesiology and Intensive Care, Université Laval, Québec, QC Canada; 6 Department of Emergency Medicine, Dalhousie University, Halifax, NS Canada; 7 Department of Internal Medicine, Université Laval, Québec, QC Canada; 8 Department of Emergency Medicine, University of Alberta, Edmonton, AB Canada; 9 Department of Family and Emergency Medicine, University of Montreal, Montreal, QC Canada; 10 Department of Emergency Medicine, Dalhousie University, Saint John, NB Canada; 11 Department of Emergency Medicine, University of Saskatchewan, Saskatoon, SK Canada; 12 Division of Emergency Medicine, Department of Medicine, Schulich School of Medicine and Dentistry, Western University and Lawson Health Research Institute, London Health Sciences Centre, London, ON Canada; 13 Centre for Clinical Epidemiology and Evaluation, Vancouver Coastal Health Research Institute, Vancouver, Canada; 14 Division of Emergency Medicine, McMaster University, Hamilton, ON Canada; 15 Department of Emergency Medicine, Queen’s University, Kingston, ON Canada

**Keywords:** Intubation, Airway management, Airway, COVID-19, Protected intubation, Patient safety, Intubation, Gestion des voies aériennes, Voies respiratoires, COVID-19, Intubation protégée, Sécurité des patients

## Abstract

**Objective:**

Intubation practices changed during the COVID-19 pandemic to protect healthcare workers from transmission of disease. Our objectives were to describe intubation characteristics and outcomes for patients tested for SARS CoV-2 infection. We compared outcomes between patients testing SARS COV-2 positive with those testing negative.

**Methods:**

We conducted a health records review using the Canadian COVID-19 Emergency Department Rapid Response Network (CCEDRRN) registry. We included consecutive eligible patients who presented to one of 47 EDs across Canada between March 1, 2020 and June 20, 2021, were tested for SARS-CoV-2 and intubated in the ED. The primary outcome was the proportion of patients experiencing a post-intubation adverse event during the ED stay. Secondary outcomes included first-pass success, intubation practices, and hospital mortality. We used descriptive statistics to summarize variables with subgroup differences examined using t tests, z tests, or chi-squared tests where appropriate with 95% CIs.

**Results:**

Of 1720 patients with suspected COVID-19 who were intubated in the ED during the study period, 337 (19.6%) tested SARS-CoV-2 positive and 1383 (80.4%) SARS-CoV-2 negative. SARS-CoV-2 positive patients presented to hospital with lower oxygen levels than SARS-CoV-2 negative patients (mean pulse oximeter SaO2 86 vs 94%, p < 0.001). In total, 8.5% of patients experienced an adverse event post-intubation. More patients in the SARS-CoV-2 positive subgroup experienced post-intubation hypoxemia (4.5 vs 2.2%, p = 0.019). In-hospital mortality was greater for patients who experienced intubation-related adverse events (43.2 vs 33.2%, p = 0.018). There was no significant difference in adverse event-associated mortality by SARS-CoV-2 status. First-pass success was achieved in 92.4% of all intubations, with no difference by SARS-CoV-2 status.

**Conclusions:**

During the COVID-19 pandemic, we observed a low risk of adverse events associated with intubation, even though hypoxemia was common in patients with confirmed SARS-CoV-2. We observed high rates of first-pass success and low rates of inability to intubate. The limited number of adverse events precluded multivariate adjustments. Study findings should reassure emergency medicine practitioners that system modifications made to intubation processes in response to the COVID-19 pandemic do not appear to be associated with worse outcomes compared to pre-COVID-19 practices.

**Supplementary Information:**

The online version contains supplementary material available at 10.1007/s43678-023-00487-1.

## Résumé

**Objectif:**

Les pratiques d'intubation ont changé au cours de la pandémie de COVID-19 afin de protéger le personnel de santé contre la transmission de la maladie. Nos objectifs étaient de décrire les caractéristiques de l'intubation et les résultats pour les patients testés pour l'infection par le CoV-2 du SRAS. Nous avons comparé les résultats entre les patients testés positifs au SARS COV-2 et ceux testés négatifs.

**Méthodes:**

Nous avons effectué un examen des dossiers de santé à l'aide du registre du Réseau canadien d'intervention rapide dans les services d’urgence pour la COVID-19 (RCIRSUC). Nous avons inclus les patients éligibles consécutifs qui se sont présentés à l'un des 47 services d'urgence du Canada entre le 1er mars 2020 et le 20 juin 2021, qui ont été testés pour le SRAS-CoV-2 et qui ont été intubés dans le service d'urgence. Le résultat principal était la proportion de patients ayant subi un événement indésirable après l'intubation pendant leur séjour aux urgences. Les critères de jugement secondaires comprenaient le succès du premier passage, les pratiques d'intubation et la mortalité hospitalière. Nous avons utilisé des statistiques descriptives pour résumer les variables avec des différences de sous-groupes examinées à l'aide de tests t, de tests z ou de tests du chi carré, le cas échéant, avec des IC à 95%.

**Résultats:**

Sur les 1720 patients suspects de COVID-19 qui ont été intubés aux urgences pendant la période de l'étude, 337 (19,6%) ont été testés positifs au SARS-CoV-2 et 1383 (80,4%) négatifs au SARS-CoV-2. Les patients positifs au SRAS-CoV-2 se sont présentés à l'hôpital avec des niveaux d'oxygène inférieurs à ceux des patients négatifs pour le SRAS-CoV-2 (oxymètre de pouls moyen SaO2 86% contre 94%, p < 0,001). Au total, 8,5% des patients ont présenté un événement indésirable après l'intubation. Un plus grand nombre de patients du sous-groupe positif au SRAS-CoV-2 ont présenté une hypoxémie post-intubation (4,5% vs 2,2%, p = 0,019). La mortalité hospitalière était plus élevée chez les patients ayant subi des événements indésirables liés à l'intubation (43,2% vs 33,2%, p = 0,018). Il n’y avait pas de différence significative dans la mortalité associée aux événements indésirables selon le statut du SRAS-CoV-2. Le succès du premier passage a été obtenu dans 92,4% de toutes les intubations, sans différence selon le statut SARS-CoV-2

**Conclusions:**

Pendant la pandémie de COVID-19, nous avons observé un faible risque d'événements indésirables associés à l'intubation, même si l'hypoxémie était fréquente chez les patients atteints de SRAS-CoV-2 confirmé. Nous avons observé des taux élevés de réussite du premier passage et des taux faibles d'incapacité à intuber. Le nombre limité d’événements indésirables a empêché les ajustements multivariés. Les résultats de l'étude devraient rassurer les praticiens de la médecine d'urgence que les modifications apportées aux processus d'intubation en réponse à la pandémie de COVID-19 ne semblent pas être associées à des résultats plus défavorables que les pratiques antérieures à la pandémie de COVID-19.

## Clinician’s capsule

**Table:** 

***What is known about the topic?***
Intubation practices changed across Canada in response to the COVID-19 pandemic to protect healthcare workers from transmission of SARS-CoV-2.
***What did this study ask?***
We described intubation characteristics and outcomes for patients with suspected and confirmed COVID-19 in EDs across Canada during the pandemic.
***What did this study find?***
We observed a low risk of intubation-associated adverse events, high rates of first-pass success and low rates of inability to intubate.
***Why does this study matter to clinicians?***
This large multicentre pan-Canadian study provides reassurance that intubation practice changes made in response to the COVID-19 pandemic appear safe and effective compared to previously published outcomes.

## Introduction

In response to the COVID-19 pandemic, healthcare systems internationally rapidly instituted changes to intubation procedures to protect healthcare workers against occupational exposure to COVID-19. The impact new protocols had on intubation practices, and patient outcomes is unknown. While we lack evidence-based national and international guidelines for COVID-19 intubations, common recommendations included enhanced personal protective equipment (PPE) for all healthcare workers attending intubations, the most experienced available providers performing intubation, video laryngoscopy, use of high-efficiency particulate air filters, and conducting intubations in negative pressure rooms with air exchangers [1–7]. This increased focus on prevention of transmission of respiratory pathogens and healthcare worker safety during emergent intubation represents a fundamental shift in the previous standard of care [1, 3, 8].

Our objectives were to describe ED intubation characteristics and outcomes for SARS-CoV-2 tested patients, and compare intubation practices, first-pass success rates, adverse events, and subsequent length of stay between patients testing SARS-CoV-2 positive with those testing negative.

## Methods

### Study design and setting

This health records review enrolled consecutive eligible patients who presented to the EDs of 47 of the 50 sites participating in the Canadian COVID-19 Emergency Department Rapid Response Network (CCEDRRN), a collaborative pan-Canadian research network (https://www.ccedrrn.com, (Supplementary Table 1)) between March 1, 2020 and June 30, 2021[9]. Three CCEDRRN sites had no patients meeting inclusion criteria at the time of data analysis. The CCEDRRN registry contains detailed clinical, laboratory and operational data on patients of all ages with suspected or confirmed COVID-19. Information on the network and our cohort, including the methodologic processes employed by the CCEDRRN, has been previously published [9–13].

### Data collection

Research assistants screened SARS-CoV-2 testing lists at all sites and manually reviewed the ED census to ensure enrolment of a complete sample, minimizing selection bias. Data extraction of eligible cases occurred via electronic medical record and/or manual review of electronic and paper charts by trained research assistants. Consecutive, eligible patients enrolled in the registry were assigned unique identifiers. Trained research assistants entered anonymized participant data into a REDCap database (V.10.9.4; Vanderbilt University, Nashville, Tennessee, USA). Reliability of health record data abstraction was confirmed through comparison to prospective data collection in a sample of patients [9]. Data quality checks were regularly performed and included verification of extreme or outlying values with oversight from a CCEDRRN coordinating centre [9, 11].

### Participants

Inclusion in this study cohort was restricted to consecutive adults [9] (17 years old) tested for SARS-CoV-2 and intubated in the ED. A confirmed case was defined as any patient with a positive SARS-CoV-2 nucleic acid amplification test during the index ED visit or within 24 h of admission to hospital, or who visited the ED with symptoms of COVID-19 and a positive SARS-CoV-2 nucleic acid amplification test within two weeks prior to the visit. This allowed us to include patients diagnosed with COVID-19 prior to their ED visit, with unavailable results, and those with early false-negative tests. We excluded patients transferred into a CCEDRRN hospital after intubation, those changed to a resuscitation status incompatible with ongoing mechanical ventilation, and patients who were misclassified as intubated.

### Waves

We defined a ‘wave’ as a period of sustained acceleration in cases followed by a period of sustained deceleration in cases on the WHO dashboard for Canada [10]: Wave 1 was the period from March 1 to June 30, 2020; wave 2 from July 1, 2020 to February 28, 2021; and wave 3 from March 1 to June 31, 2021.

### Outcomes

Our primary outcome was the proportion of patients experiencing a post-intubation adverse event in the ED. Adverse events included hypoxemia (SpO2 < 80%), vomiting, arrhythmias, cardiac arrest, dental trauma, the need for a surgical airway or inability to establish an airway. Secondary outcomes included the proportion of patients with first-pass success (as documented in the health record), intubation practices (pre-oxygenation methods, use of bag-mask ventilation, sedative and/or paralytic use, mode of laryngoscopy), and clinical outcomes (in-hospital mortality, and Intensive Care Unit (ICU) and hospital length of stay).

### Analysis

Assuming a proportion of post-intubation adverse events of at most 0.2, a sample size of 800 would provide a 95% confidence interval (CI) for a proportion of width at most 0.057. Descriptive statistics (means, standard deviations (SDs), medians, inter-quartile ranges (IQRs) and proportions) were used to summarize variables. Differences between groups of patients defined by SARS-CoV-2 test results were examined using t-tests, z-tests, or chi-squared tests where appropriate with associated 95% CIs. We planned a multivariable logistic regression model to examine factors associated with adverse events but encountered insufficient adverse events to complete this analysis. To protect participant privacy, we utilized a cell restriction policy and did not report counts < 5. We considered p values (p) < 0.05 statistically significant. We did not adjust for multiple comparisons. All analyses were conducted in R (2021, Vienna, Austria).

### Patient and public engagement

A dedicated Patient Engagement Committee with patient partners from across Canada advise the network, and provide input into research questions, study design and outcomes, data interpretation, manuscript development and knowledge translation for studies. Patient partners sit on CCEDRRN’s Executive, Scientific Advisory, Protocol Review & Publications, Data Access & Monitoring, and Knowledge Translation Committees.

### Ethics

Data collection within the CCEDRRN registry was approved by the research ethics boards of record for all participating sites.

## Results

**Fig. 1:**
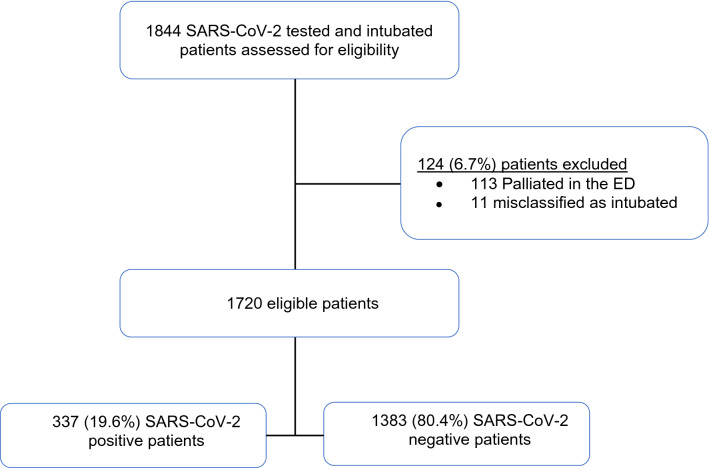
Participant flow diagram

We identified 1844 consecutive SARS-CoV-2-tested adults who were intubated in a CCEDRRN ED during the study period (Fig. 1). After excluding 113 patients who experienced changes in their goals of care in the ED precluding mechanical ventilation, and 11 individuals misclassified as intubated in the registry, we included 1720 patients. Of these, 337 (19.6%) tested positive and 1383 (80.4%) negative for SARS-CoV-2.

### Baseline characteristics

Our cohort included patients from 7 provinces across Canada. 465 (27.0%) presented in the first, 831 (48.3%) in the second and 424 (24.7%) in the third wave. Patients with SARS-CoV-2 intubated in EDs were older than SARS-CoV-2 negative patients intubated in EDs (median 62 years vs. 58 years, p < 0.001). At the time of triage, SARS-CoV-2 positive patients had higher mean respiratory rates (32 bpm vs. 23 bpm, p < 0.001), and lower mean oxygen saturations (86% vs. 94%, p < 0.001) compared to SARS-CoV-2 negative patients; additionally, 25.3% of SARS-CoV-2 positive patients had a pulse oximetry SaO2 < 80% at triage compared to 5.2% of those testing negative (p < 0.001). SARS-CoV-2 positive patients tended to have more high flow oxygen via nasal cannula (16 vs. 1.5%, p < 0.001) and non-invasive ventilation (8.9 vs. 3.5%, p < 0.001) as oxygen support modalities prior to the need for intubation. They also had higher mean arrival Glasgow Coma Scores (GCS) (12 vs. 8, p < 0.001). (Table 1).

**Table 1:** Baseline Characteristics of Patients with and without SARS-CoV-2

Variable	SARS-CoV-2 + (n, %) (337, 19.6)	SARS-CoV-2 – (n, %) (1383, 80.4)
Province, n (%)		
Alberta	118 (35.0)	128 (9.3)
British Columbia	113 (33.5)	713 (51.5)
New Brunswick	< 5	< 5
Nova Scotia	< 5	7 (0.5)
Ontario	30 (8.9)	260 (18.8)
Quebec	71 (21.1)	201 (14.5)
Saskatchewan	< 5	72 (0.6)
Age in years, median (IQR)	62.0 (52.0,72.0)	57.6 (40.0,69.0)
Female, n (%)	118 (35.0)	453 (32.8)
Pandemic wave		
Wave 1 (March 1,2020—June 30, 2020)	54 (16.2)	411 (29.7)
Wave 2 (July 1, 2020 – February 28, 2021)	166 (49.2)	665 (48.1)
Wave 3 (March 1, 2021 – June 31, 2021)	117 (34.7)	307 (22.2)
Comorbidities, n (%)		
Hypertension	116(34.4)	409(29.6)
Psychiatric condition	33(9.8)	305(22.1)
Diabetes	74(22.0)	204(14.8)
Dyslipidemia	63(18.7)	193(14.0)
Chronic neurological disorder	25(7.4)	183(13.2)
Chronic lung disease	27(8.0)	128(9.3)
Coronary artery disease	23(6.8)	126(9.1)
Rheumatologic disorder	25(7.4)	119(8.6)
Atrial fibrillation	9(2.7)	87(6.3)
Chronic kidney disease	16(4.8)	77(5.6)
Substance Use, n (%)		
Smoking/vaping	44(13.1)	296(21.4)
Illicit substance use	33(9.8)	323(23.4)
Arrival From, n (%)		
Home	297(88.1)	1049(75.8)
Unstable housing	16(4.8)	101(7.3)
Long term care/rehab	10(3.0)	49(3.5)
Inter-facility transfer	7(2.1)	161(11.6)
Correctional facility	< 5	< 5
Canadian triage and acuity scale, n (%)		
1	214(63.5)	984(71.4)
2	100(29.7)	321(23.3)
3	20(5.9)	67(4.9)
4	< 5	5
5	< 5	< 5
Arrival vital signs		
Heart Rate (BPM), mean (SD)	107 (25.6)	98 (28.7)
Systolic BP, mean (SD)	128 (27.5)	130 (35.9)
Diastolic BP, mean (SD)	75 (17.0)	77 (22.8)
RR, mean (SD)	32 (11.7)	23 (9.3)
Pulse oximetry SpO2, mean (SD)	86 (13.7)	94 (8.5)
Temperature (Celsius), mean (SD)	37 (2.7)	36 (1.4)
Glasgow Coma Score, mean (SD)	12 (4.5)	8 (4.8)
Oxygen delivery prior to intubation, n (%)		
Nasal prongs	38 (11.3)	158 (11.4)
Facemask/Rebreather/Non-Rebreather	98 (29.1)	284 (20.5)
High-flow nasal cannulae	54 (16.0)	21 (1.5)
CPAP, non-invasive ventilation	30 (8.9)	47 (3.5)

*SD* standard deviation, *IQR*: interquartile range represented as 25th percentile, 75th percentile, *BPM* beats per minute, *BP* blood pressure, *RR* respiratory rate, *SpO2* pulse oximetry, *CPAP*: continuous positive airway pressure

### Characterizing ED intubation during COVID-19

With respect to pre-oxygenation, there was significantly less bag-mask ventilation used in the group of patients testing positive for SARS-CoV-2 compared to those testing negative (14.2 vs. 26.5%, p < 0.001). These SARS-CoV-2 positive patients were more likely to be pre-oxygenated with humidified oxygen via high-flow nasal cannulae (HFNC) (15.4 vs. 1.1%, p < 0.001), non-invasive ventilation (8.0 vs. 3.0%, p < 0.001) and/or nasal prongs/face mask (37.1 vs. 29.1%, p = 0.005) than SARS-CoV-2 negative patients. Propofol was less commonly used as an induction agent in SARS-CoV-2 positive patients (45.7 vs. 55.2%, p = 0.002). Paralytic use was documented in 260/274 (95%) of intubations for SARS-CoV-2 positive patients and 908/993 (91%) of intubations for patients who were SARS-CoV-2 negative. There was no evidence of differences in laryngoscopy methods between groups (Table 2).

**Table 2:** Intubation practices in patients with and without SARS-CoV-2

Intubation practices	SARS-CoV-2 + (n, %) (337, 19.6)	SARS-CoV-2 – (n, %) (1383, 80.4)
Pre-oxygenation method		
Bag Mask Ventilation	48 (14.2)	366 (26.5)
High Flow Nasal Cannulae	52 (15.4)	15 (1.1)
Non-Invasive Ventilation	27 (8.0)	41 (3.0)
Nasal Prong/Face Mask	125 (37.1)	403 (29.1)
None	< 5	56 (4.1)
Sedative used		
Ketamine	174(51.6)	637(46.1)
Etomidate	12(3.6)	40(2.9)
Dexmedetomidine	< 5	< 5
Midazolam	40 (11.9)	129(9.3)
Fentanyl	60 (17.8)	233(16.9)
Propofol	154 (45.7)	763(55.2)
Paralytic Used		
NDMBs	208 (52.8)	730 (61.7)
DMBs	52 (15.4)	178 (12.9)
None	14 (4.2)	85 (6.2)
Not documented	12 (3.6%)	26 (1.9%)
Intubation technique/laryngoscopy		
Video laryngoscopy	209 (62.0)	884 (63.9)
Direct laryngoscopy	42 (12.5)	132 (9.5)
Fibre-optic	5 (1.5)	9 (0.7)
Blind nasotracheal	< 5	8 (0.6)
Other	15 (4.5)	81 (5.9)
Not documented	90 (26.7)	360 (26.0)

*NDMBs* non-depolarizing neuromuscular blockers, *DMBs* depolarizing neuromuscular blockers

### Adverse events

In total, 147/1720 (8.5%) patients experienced an adverse event post-intubation. Significantly more SARS-CoV-2 positive patients experienced hypoxemia (8.9 vs. 3.2%, p < 0.001) compared with negative patients. There were no recorded events of inability to establish an airway (Table 3). Though in-hospital mortality was greater for patients who experienced adverse events at the time of intubation (43.2 vs. 33.2%, p = 0.018), there was no significant difference in mortality between SARS-CoV-2 positive patients with and without adverse events (23.3 vs. 20.5%, p = 0.613) or between SARS-CoV-2 negative patients with and without adverse events (76.7 vs. 79.5%, p = 0.613). Median ICU length of stay was greater for patients with adverse events (9 days vs. 6 days, p = 0.019), with SARS-CoV-2 positive patients having longer ICU length of stay (p = 0.009) than those testing negative. SARS-CoV-2 positive patients had longer median hospital length of stay irrespective of exposure to an adverse event (15 days). There was no significant difference in median hospital length of stay between patients with and without adverse events (8 days vs. 10 days, p = 0.658) (Supplementary Table 2).

**Table 3:** Intubation Outcomes in Patients with and without SARS-CoV-2

Intubation Outcomes	SARS-CoV-2 + (n, %) (337, 19.6)	SARS-CoV-2 – (n, %) (1383, 80.4)
Adverse events		
Hypoxemia (SpO2 < 80%)	30 (8.9)	44 (3.2)
Vomiting	< 5	19 (1.4)
Arrhythmia	< 5	< 5
Cardiac arrest	7 (2.1)	39 (2.8)
Dental trauma	< 5	< 5
Surgical airway	< 5	< 5
Unable to establish airway	< 5	< 5
First-pass success	313 (92.9)	1269 (91.8)
Second pass	19 (5.6)	89 (6.4)
Third pass	< 5	18 (1.3)
More than third pass	< 5	7 (0.5)
Rescue device	17(5.0)	72 (5.2)
Unable to establish airway	0	0

*SpO2* pulse oximetry

### First-pass success

In our cohort, 1582/1712 (92.4%) patients were intubated with first-pass success. There were no differences between the proportion of SARS-CoV-2-positive and negative patients (92.9 vs. 91.8%, p = 0.497) (Table 3). There were no differences for in-hospital mortality, ICU or hospital length of stay for patients intubated with first-pass success compared to those requiring multiple attempts (Supplementary Table 3).

## Discussion

### Interpretation

This is the largest study characterizing intubation practices in Canadian EDs during the first three waves of the COVID-19 pandemic. By characterizing patients tested for SARS-CoV-2 requiring intubation in EDs and evaluating relevant outcomes, we have added insights into emergency airway management during the pandemic. Reassuringly, we did not observe differences in first-pass success in relation to SARS-CoV-2 status, and first-pass success was not associated with differences in mortality or length of stay. Patients with SARS-CoV-2 experienced proportionally more intubation-related hypoxemic events compared to those who were SARS-CoV-2 negative, but also presented more frequently with hypoxemia at baseline. While in-hospital mortality was higher in patients who experienced adverse events associated with intubation, these results could not be adjusted for baseline hypoxemia due to low event rates. We found no association between SARS-CoV-2 status and mortality regardless of adverse events.

### Previous studies

High rates of first-pass success among patients with and without SARS-CoV-2 are consistent with first-pass success rates from resource-rich settings, [4–7] and notably better than first-pass success rates globally prior to the onset of the COVID-19 pandemic [14]. Existing published reports of intubation processes and outcomes during the COVID-19 pandemic are limited to small single-centre studies [15–21]. Of these, only two studies described intubations performed exclusively by emergency physicians with first-pass-success rates ranging from 82% [16] to 91% [19]. In early studies during the COVID-19 pandemic fewer intubators were emergency physicians (as compared to anesthesiologists or critical care medicine physicians), and first-pass-success ranged from 86% [17] to 89% [18]. In contrast to these early studies, our larger cohort included 3 waves of the pandemic and our observed first-pass-success rate of > 92% provides reassurance of safety in airway management processes changes made across Canada in response to COVID-19 and serves as a testament to the airway expertise in the Canadian EM community. The impact of intubator experience may have played a role in the high rate of first-pass success observed (as many position statements recommended the most experienced available provider perform intubations) but this variable was not available in the CCEDRRN registry. Studies comparing pre- and post-COVID-19 intubation practices found intubator experience to be positively associated with first-pass success [22]. First-pass success is an operational surrogate for patient outcomes [23], with multiple intubation attempts having been associated with increased adverse events and complications [4, 23, 24]. Expectedly, we found patients with SARS-CoV-2 experienced more hypoxemia following intubation than those without SARS-CoV-2 with no differences in first-pass success rates. While these events were counted as adverse events, baseline hypoxemia likely confounded these results, with adverse events being too infrequent for meaningful adjusted analyses. This finding highlights the limitation of first-pass success as a surrogate for post-intubation adverse events, and this imperfect association should be accounted for in future research on intubation of patients who are hypoxic at baseline.

There have been limited evaluations of adverse events related to intubation during the COVID-19 pandemic from small 1–2 centre cohorts [15–20]. The reported proportions of patients experiencing post-intubation hypoxemia during the COVID-19 pandemic ranges widely from 8% [17] to 73% [20] suggesting wide variation in patient populations, pre-oxygenation or intubation practices. In our nationally representative large cohort, we found low rates of post-intubation hypoxemia (4%) overall, including in SARS-CoV-2 positive patients (9%). This supports the hypothesis that changes made to intubation process in EDs to protect healthcare workers may not have adversely impacted patient safety. In our study, patients with and without SARS-CoV-2 likely had different indications for intubation. Patients with SARS-CoV-2 likely required intubation for hypoxic respiratory failure compared to SARS-CoV-2 negative patients who presumably had heterogenous indications for intubation, although available data lacked sufficient granularity to evaluate this explicitly.

### Strengths and limitations

This study was observational and retrospective. Secondary outcomes and subgroup analyses should be considered hypothesis generating. Data collected was dependent on documented intubation events, which could have been underreported. Despite a nationally representative sample, most EDs were urban, and managed a high number of acutely sick COVID-19 patients. Observations may be less reflective of rural practice. For many patients, unmeasured and measured confounders and co-interventions that may influence mortality were not captured in our registry and we could not account for in multivariable analysis due to the low number of adverse outcomes. Thus, causal relationships cannot be inferred. We did not adjust p-values for multiple comparisons so statistically significant differences should be considered hypothesis generating.

### Clinical implications

ED practitioners can continue using modified intubation techniques that include an enhanced focus on healthcare worker safety and be reassured that these practices do not appear to be associated with worse patient outcomes compared to pre-COVID-19 practices. Specific elements of intubation training and quality improvement (e.g., the use of in situ simulation training, briefing checklists, etc.) should be informed by future research.

### Research implications

Further research is needed to understand whether intubation-related adverse events are causally associated with increased morbidity or mortality and to understand the independent effects of the multi-component intubation process interventions that were employed in response to the COVID-19 pandemic. Indication for- and optimal timing of intubation and invasive mechanical ventilation for patients with COVID-19 pneumonia and respiratory failure remain unknown.

## Conclusions

During the first three waves of the COVID-19 pandemic, we observed high rates of first-pass success and low rates of failed airways. We observed a low risk of adverse events overall, with higher rates of hypoxia among patients testing positive for SARS CoV-2 compared to those who tested negative. The findings of this study provide reassurance that system modifications made to intubation processes in response to the COVID-19 pandemic do not appear to be associated with worse outcomes compared to pre-COVID-19 practices.

## Supplementary Information

Below is the link to the electronic supplementary material.

Supplementary file1 (DOCX 43 KB)

## Data Availability

The data that support these findings are not openly available due to reasons of sensitivity but are available from the CCEDRRN upon reasonable request (www.ccedrrn.com).

## References

[1] Janz DR, Semler MW, Joffe AM (2018). A multicenter randomized trial of a checklist for endotracheal intubation of critically ill adults. Chest.

[2] Brewster DJ, Chrimes N, Do TB (2020). Consensus statement: safe airway society principles of airway management and tracheal intubation specific to the <scp>COVID</scp> -19 adult patient group. Med J Aust.

[3] Brindley PG, Beed M, Duggan LV, Hung O, Murphy MF (2016). Updating our approach to the difficult and failed airway: time to “stop and think”. Can J Anesth Can d’anesthésie.

[4] Park L, Zeng I, Brainard A (2017). Systematic review and meta-analysis of first-pass success rates in emergency department intubation: creating a benchmark for emergency airway care. Emerg Med Australas.

[5] Phillips L, Orford N, Ragg M (2014). Prospective observational study of emergent endotracheal intubation practice in the intensive care unit and emergency department of an Australian regional tertiary hospital. Emerg Med Australas.

[6] Ferguson I, Buttfield A, Burns B, Reid C, Shepherd S, Milligan J, Harris IA, Aneman A (2022). Fentanyl versus placebo with ketamine and rocuronium for patients undergoing rapid sequence intubation in the emergency department: the <scp>FAKT</scp> study—A randomized clinical trial. Acad Emerg Med.

[7] Brown CA, Bair AE, Pallin DJ, Walls RM (2015). Techniques, success, and adverse events of emergency department adult intubations. Ann Emerg Med.

[8] Higgs A, McGrath BA, Goddard C, Rangasami J, Suntharalingam G, Gale R, Cook TM (2018). Guidelines for the management of tracheal intubation in critically ill adults. Br J Anaesth.

[9] Hohl CM, Rosychuk RJ, McRae AD (2021). Development of the Canadian COVID-19 emergency department rapid response network population-based registry: a methodology study. C Open.

[10] Hohl CM, Rosychuk RJ, Hau JP (2022). Treatments, resource utilization, and outcomes of COVID-19 patients presenting to emergency departments across pandemic waves: an observational study by the Canadian COVID-19 emergency department rapid response network (CCEDRRN). Can J Emerg Med.

[11] McRae AD, Hohl CM, Rosychuk R (2021). CCEDRRN COVID-19 Infection Score (CCIS): development and validation in a Canadian cohort of a clinical risk score to predict SARS-CoV-2 infection in patients presenting to the emergency department with suspected COVID-19. BMJ Open.

[12] Hohl CM, Rosychuk RJ, Archambault PM (2022). The CCEDRRN COVID-19 Mortality Score to predict death among nonpalliative patients with COVID-19 presenting to emergency departments: a derivation and validation study. C Open.

[13] Davis P, Rosychuk R, Hau JP (2022). Diagnostic yield of screening for SARS-CoV-2 among patients admitted to hospital for alternate diagnoses: an observational cohort study. BMJ Open.

[14] Russotto V, Myatra SN, Laffey JG (2021). Intubation practices and adverse peri-intubation events in critically ill patients from 29 countries. JAMA.

[15] Ahmad I, Jeyarajah J, Nair G, Ragbourne SC, Vowles B, Wong DJN, El-Boghdadly K (2021). A prospective, observational, cohort study of airway management of patients with COVID-19 by specialist tracheal intubation teams. Can J Anesth Can d’anesthésie.

[16] de Alencar JCG, Marques B, Marchini JFM (2020). First-attempt intubation success and complications in patients with COVID-19 undergoing emergency intubation. J Am Coll Emerg Physicians Open.

[17] Dullemond K, Renschler C, Trojanowski J, Scheuermeyer F, Stenstrom R, Griesdale D, MacRedmond R, Nattrass E, Farina L, Yoo J (2021). Success and complications of endotracheal intubation in critical care settings under COVID-19 protocols. Can J Emerg Med.

[18] Hawkins A, Stapleton S, Rodriguez G, Gonzalez RM, Baker W (2021). Emergency tracheal intubation in patients with COVID-19: a single-center, retrospective cohort study. West J Emerg Med.

[19] Soh M, Hifumi T, Otani N (2022). Trends in endotracheal intubation for patients with COVID-19 by emergency physicians. Glob Heal Med.

[20] Yao W, Wang T, Jiang B (2020). Emergency tracheal intubation in 202 patients with COVID-19 in Wuhan, China: lessons learnt and international expert recommendations. Br J Anaesth.

[21] Zhang L, Li J, Zhou M, Chen Z (2020). Summary of 20 tracheal intubation by anesthesiologists for patients with severe COVID-19 pneumonia: retrospective case series. J Anesth.

[22] Black H, Hall T, Hrymak C (2022). A prospective observational study comparing outcomes before and after the introduction of an intubation protocol during the COVID-19 pandemic. Can J Emerg Med.

[23] Sakles JC, Chiu S, Mosier J, Walker C, Stolz U (2013). The importance of first pass success when performing orotracheal intubation in the emergency department. Acad Emerg Med.

[24] De Jong A, Rolle A, Pensier J, Capdevila M, Jaber S (2020). First-attempt success is associated with fewer complications related to intubation in the intensive care unit. Intensive Care Med.

