# Supplementary material for: Intubation practices and outcomes for patients with suspected or confirmed COVID-19: a national observational study by the Canadian COVID-19 Emergency Department Rapid Response Network (CCEDRRN)
Source: CJEM. 2023 Apr 5;25(4):335–43. doi: 10.1007/s43678-023-00487-1 (PMC10075161; doi:10.1007/s43678-023-00487-1)
Supplement: Supplementary file 1 — Supplementary file1 (DOCX 43 KB) [file 43678_2023_487_MOESM1_ESM.docx]

**Appendix**

*Appendix Table 1 - Canadian COVID-19 Emergency Department Rapid Response Network (CCEDRRN) Participating Recruitment Sites and Patient Enrollment.*

| **Site Name** | **Province** | **Start Date** | **End Date** | **Number of Patients** |
| --- | --- | --- | --- | --- |
| Vancouver General Hospital | British Columbia | 2020-03-01 | 2021-11-30 | 579 |
| Saint Paul's Hospital | British Columbia | 2020-03-01 | 2021-06-21 | 107 |
| Mount Saint Joseph's | British Columbia | 2020-03-01 | 2021-03-24 | 0 |
| Lions Gate Hospital | British Columbia | 2020-03-01 | 2021-04-30 | 12 |
| Surrey Memorial Hospital | British Columbia | 2020-03-19 | 2021-04-30 | 52 |
| Royal Columbian Hospital | British Columbia | 2020-03-01 | 2021-05-31 | 34 |
| Eagle Ridge | British Columbia | 2020-03-01 | 2021-04-30 | 10 |
| Abbottsford Regional Hospital | British Columbia | 2020-04-20 | 2021-07-15 | 25 |
| Kelowna General Hospital | British Columbia | N/A | N/A | 5 |
| Royal Inland Hospital | British Columbia | N/A | N/A | <5 |
| Hôtel-Dieu de Lévis | Quebec | 2020-05-04 | 2021-05-18 | <5 |
| Jewish General Hospital | Quebec | 2020-03-01 | 2021-06-30 | 103 |
| Hôpital de l'Enfant-Jésus (CHU de Québec) | Quebec | 2020-05-04 | 2021-08-08 | 129 |
| Centre Hospitalier de l'Université Laval | Quebec | N/A | N/A | <5 |
| Hôpital du Saint-Sacrement | Quebec | N/A | N/A | <5 |
| Hôpital Saint-François d'Assise | Quebec | N/A | N/A | <5 |
| Hôtel-Dieu de Québec (CHU de Québec) | Quebec | N/A | N/A | <5 |
| L'hôpital Royal Victoria (MUHC) | Quebec | 2020-05-04 | 2021-05-11 | 10 |
| Montréal General Hospital (MUHC) | Quebec | 2020-05-04 | 2021-05-06 | <5 |
| Hôpital du Sacré-Coeur | Quebec | 2020-05-04 | 2021-05-18 | 18 |
| IUCPQ: Institut universitaire de cardiologie et de pneumologie de Québec | Quebec | 2020-05-04 | 2021-05-13 | <5 |
| Sunnybrook | Ontario | 2020-05-14 | 2021-02-03 | 76 |
| University Health Network - Toronto Western Hospital | Ontario | 2020-09-01 | 2021-12-31 | 53 |
| The Ottawa Hospital - Civic Campus | Ontario | 2020-05-14 | 2021-05-31 | 11 |
| The Ottawa Hospital - General Campus | Ontario | 2020-05-14 | 2021-05-18 | <5 |
| Kingston General Hospital | Ontario | 2020-05-14 | 2021-08-10 | 14 |
| Hotel Dieu Hospital, Kingston | Ontario | 2020-05-14 | 2021-06-14 | 0 |
| Hamilton Health Sciences - Hamilton General Hospital | Ontario | N/A | N/A | <5 |
| Hamilton Health Sciences - Juravinski Hospital | Ontario | N/A | N/A | <5 |
| Health Science North, Sudbury Ontario | Ontario | 2020-05-14 | 2021-12-31 | 124 |
| London Health Sciences Centre | Ontario | N/A | N/A | 5 |
| North York General Hospital Toronto | Ontario | 2020-05-14 | 2020-06-04 | 0 |
| University of Alberta Hospital, Edmonton | Alberta | 2020-04-08 | 2021-05-07 | 19 |
| Foothills, Calgary | Alberta | 2020-03-01 | 2021-04-07 | 37 |
| Rockyview, Calgary | Alberta | 2020-03-01 | 2021-04-07 | 11 |
| Peter Lougheed Centre | Alberta | 2020-03-01 | 2021-12-31 | 113 |
| South Health Campus, Calgary | Alberta | 2020-03-01 | 2021-12-31 | 37 |
| Royal Alexandra Hospital, Edmonton | Alberta | N/A | N/A | 26 |
| Northeast Community Health Centre, Edmonton | Alberta | N/A | N/A | <5 |
| St Paul’s Hospital, Saskatoon | Saskatchewan | 2020-03-01 | 2021-04-30 | <5 |
| Royal University, Saskatoon | Saskatchewan | 2020-03-17 | 2021-12-07 | 57 |
| Saskatoon City Hospital, Saskatoon | Saskatchewan | 2020-03-01 | 2021-04-30 | <5 |
| Health Sciences Centre | Manitoba | N/A | N/A | 9 |
| Saint John Regional Hospital | New Brunswick | 2020-03-12 | 2021-04-12 | <5 |
| Halifax Infirmary | Nova Scotia | 2020-04-05 | 2021-04-15 | 6 |
| Dartmouth General Hospital | Nova Scotia | 2020-04-05 | 2021-04-15 | <5 |
| Hants Community Hospital | Nova Scotia | 2020-04-05 | 2021-04-15 | <5 |
| Cobequid Community Health Centre | Nova Scotia | 2020-04-05 | 2021-04-15 | 0 |
| Secondary Assessment centers | Nova Scotia | 2020-03-26 | 2020-05-15 | 0 |

*Appendix Table 2: Comparison of Cases with Post-Intubation Adverse Events vs. No Adverse Events*

| **Outcomes** | **Adverse Events**  **n=147 (8.5%)** | **No Adverse Events**  **n=1573 (91.5%)** | **p-value** |
| --- | --- | --- | --- |
| In-Hospital Mortality (n, %)  SARS-CoV-2 +  SARS-CoV-2 - | 60/139 (43.2)  14/60 (23.3)  46/60 (76.7) | 497/1495 (33.2)  102/497 (20.5)  395/497 (79.5) | 0.018  0.613 |
| ICU LOS in days (median, IQR)  SARS-CoV-2 +  SARS-CoV-2 - | 9 (3,9)  17.5 (9.3,25)  5 (2,12) | 6 (2,13)  9 (4,17)  5 (2,12) | 0.019  0.009 |
| Hospital LOS in days (median, IQR)  SARS-CoV-2 +  SARS-CoV-2 - | 8 (1,20)  15(2.5,35)  5 (1,18) | 10 (2,23)  15(6,27)  8(2,21.5) | 0.658  0.014 |

*ICU: intensive care unit; LOS: length of stay; IQR: inter-quartile range*

*Appendix Table 3: Comparison of Cases with First-pass Success vs. Multiple Attempts*

| **Outcomes** | **First-pass Success**  **n=1582 (92.0%)** | **Multiple Attempts**  **n=130 (7.5%)** | **p-value** |
| --- | --- | --- | --- |
| In-Hospital Mortality (n, %)  SARS-CoV-2 +  SARS-CoV-2 - | 509/1504 (33.8)  105/509 (20.1)  404/509 (79.4) | 46/122 (37.7)  10/46 (21.7)  36/46 (78.3) | 0.387  0.859 |
| ICU LOS in days (median, IQR)  SARS-CoV-2 +  SARS-CoV-2 - | 6 (2,13)  10 (4,18)  5 (2,12) | 6 (3,18.3)  15.5 (5.3,26.5)  6(3,15) | 0.770  0.471 |
| Hospital LOS in days (median, IQR)  SARS-CoV-2 +  SARS-CoV-2 - | 9 (2,22)  15 (5,27.5)  8 (2,21) | 9.5 (2.3,24)  15 (9.5,34)  8 (2,23) | 0.595  0.728 |

*ICU: intensive care unit; LOS: length of stay; IQR: interquartile range represented as 25^th^ percentile, 75^th^ percentile*
